# Supplementary material for: Comprehensive Annotation and Functional Exploration of MicroRNAs in Lettuce
Source: Front Plant Sci. 2021 Dec 24;12:781836. doi: 10.3389/fpls.2021.781836 (PMC8739914; doi:10.3389/fpls.2021.781836)
Supplement: Supplementary file 2 [file Data_Sheet_8.ZIP › GSM2230747.plot/Lsa-miRN45_Lsat_1_v5_gn_1_29421.1_354_TPlot.pdf]

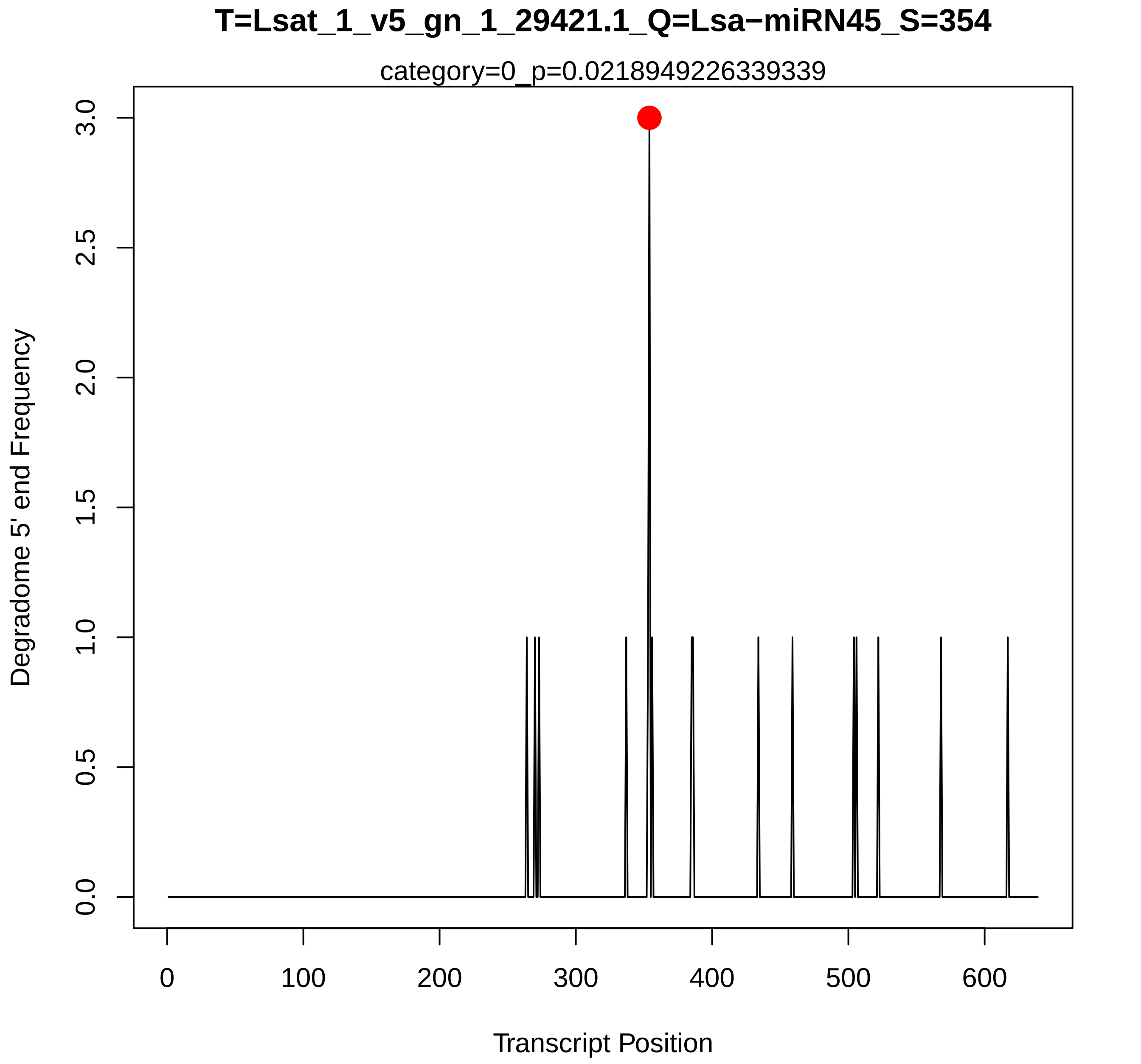

T=Lsat_1_v5_gn_1_29421.1_Q=Lsa−miRN45_S=354
category=0_p=0.0218949226339339
Degradome 5' end Frequency
0.0
0.5
1.0
1.5
2.0
2.5
3.0
0
100
200
300
400
500
600
Transcript Position
